# Long-term efficacy of vildagliptin in patients with type 2 diabetes undergoing hemodialysis

**DOI:** 10.1186/s40200-015-0214-6

**Published:** 2015-11-05

**Authors:** Jun-ichiro Mera, Eiko Okada, Masumi Okuda, Tatsuru Ota, Shigeru Sibata, Shunya Uchida

**Affiliations:** Department of Hemodialysis, Shinsen Ikebukuro Clinic, Tobu Annex Bldg. 4F, 1-10-10, Nishi-Ikebukuro, Toshima-ku, Tokyo, 171-0021 Japan; Department of Urology, Higashi-Omiya General Hospital, 5-18, Higashi-Omiya, Minuma-ku, Saitama, 337-0051 Japan; Department of Nephrology, Bousei Hospital, 1-8-14, Harigaya, Urawa-ku, Saitama, 330-0075 Japan; Department of Nephrology, Teikyo University School of Medicine, 2-11-1, Kaga, Itabashi-ku, Tokyo, 173-8606 Japan

**Keywords:** Vildagliptin, Hemodialysis, Type 2 diabetes, Glycated albumin

## Abstract

**Background:**

There are few studies evaluating long-term glycemic control using a dipeptidyl peptidase-4 inhibitor in type 2 diabetes patients with end-stage renal disease (ESRD). The aim of this study was to evaluate the safety and efficacy of vildagliptin therapy over 2 years in type 2 diabetes with ESRD.

**Methods:**

Patients with ESRD resulting from type 2 diabetes requiring dialysis who had ≥20 % glycated albumin (GA) were enrolled. Vildagliptin 50 mg once daily was administered for 2 years. Changes in GA and dry weight were evaluated.

**Results:**

In 32 patients (24 men and 8 women) aged 68.3 ± 1.9 years, vildagliptin 50 mg once daily was administered for 2 years, but the dose was increased to 50 mg twice daily in 15 patients. GA was significantly reduced by 2.6 ± 0.6 %, from 22.4 ± 0.6 % at baseline to 19.8 ± 0.4 % at 2 years. After 2 years of vildagliptin therapy, 15 (46.9 %) of 32 patients achieved a GA level of <20 %. Dry weight changed slightly, with an increase of 1.3 ± 0.8 kg at 2 years. No adverse drug reactions related to treatment with vildagliptin were seen.

**Conclusions:**

Vildagliptin is a promising therapeutic option for safe, effective glycemic control in type 2 diabetic patients with ESRD.

## Background

Diabetes is a risk factor for chronic kidney disease, and strict glycemic control in the management of diabetes can slow the progression of nephropathy [1]. Nonetheless, the number of patients with end-stage renal disease (ESRD) requiring hemodialysis is increasing linearly in Japan, and diabetic nephropathy accounts for ≥40 % of the underlying disease [2]. Therefore, prevention of the progression of chronic kidney disease in diabetic patients is a clinical and social problem that must be addressed. On the other hand, it has been reported that persistent hyperglycemia is also correlated with increased mortality in type 2 diabetic patients with ESRD, and strict glycemic control contributes to reduced mortality in this group [3].

However, since glucose metabolism is modified by renal function, hypoglycemia often occurs in the treatment of diabetic patients with ESRD. Hypoglycemia is a risk factor for cardiovascular disease as well as dementia, and the importance of avoiding hypoglycemia in antidiabetic therapy was reconfirmed by the results of the ACCORD study [4]. Nine categories of drugs, including insulin and glucagon-like peptide-1 receptor agonists, are available for the treatment of type 2 diabetes. However, since the pharmacokinetics of these drugs are affected by renal function, their use is limited in diabetic patients with ESRD [5]. Only insulin and some oral antidiabetic agents are available for the treatment of these patients, often complicating the process of treatment selection.

Dipeptidyl peptidase-4 (DPP-4) inhibitors lower blood glucose by enhancing the effect of incretin to stimulate insulin secretion in a glucose-dependent manner as well as by inhibiting paradoxical glucagon secretion [6]. Despite their recent clinical introduction, DPP-4 inhibitors, which are associated with a lower risk of drug-induced weight gain and a low incidence of hypoglycemia, now play a central role in the treatment of type 2 diabetes mellitus [7]. DPP-4 inhibitors can be administered in the presence of ESRD, and it is believed that their long-term use is safe.

In a pharmacokinetic study of the DPP-4 inhibitor vildagliptin in patients with renal dysfunction, the exposure level increased with the severity of renal impairment compared with that in healthy individuals, and therefore the dose level of 50 mg once daily was recommended for patients with renal dysfunction [8]. Although the standard dose of vildagliptin is 50 mg twice daily, vildagliptin significantly reduced HbA1c at a dose of 50 mg once daily in a Japanese study [9]. These findings indicate that vildagliptin can be used in patients with ESRD if the dose is adjusted.

Since it is not appropriate to evaluate efficacy on a short-term basis alone, the present study was designed to evaluate the safety and efficacy of vildagliptin-based therapy over 2 years in a prospective, open-label study. To avoid hypoglycemia, previous drugs were replaced with vildagliptin 50 mg once daily as the initial dose. In addition, the glycated albumin (GA) level was used as the parameter of blood glucose control instead of HbA1c, which is an informative measure of blood glucose levels but may underestimate glycemic control in patients with renal dysfunction [10].

## Methods

### Patients

Type 2 diabetic patients receiving hemodialysis 3 times a week in Shinsen Ikebukuro Clinic, during the period from April 2010 to March 2011 were included in the study. Patients who met all of the following criteria were eligible for study enrollment: 1) ESRD requiring dialysis resulting from type 2 diabetes; 2) a stable dry weight after ≥3 months of hemodialysis; 3) aged ≥40 years; 4) a GA level of ≥20 % despite ≥6 months of diet and exercise therapy or hypoglycemic therapy; and 5) ≥6 months since the initiation of insulin therapy if applicable. Those who met any of the following criteria were excluded from the study: 1) type 1 diabetes; 2) history of ketoacidosis; 3) severe hepatic dysfunction; 4) cardiovascular disease within the past 6 months; and 5) malignant tumor.

This study was approved by the institutional review boards of Shinsen Ikebukuro Clinic, and all patients provided written informed consent for study participation after explanations for the necessity of vildagliptin therapy were given. The study was conducted as an investigator-initiated study in accordance with the Declaration of Helsinki and the Ethical Guidelines for Clinical Research established by the Japanese Ministry of Health, Labor and Welfare [11].

### Study design

This was a prospective, open-label study conducted to evaluate glycemic control and the safety of vildagliptin therapy in type 2 diabetes patients with ESRD over 2 years. After it was confirmed that each patient had a stable GA level and dry weight (body weight just after hemodialysis), vildagliptin therapy was initiated at a dose of 50 mg once daily after breakfast. Vildagliptin therapy at a dose of 50 mg twice daily (after breakfast and dinner) was also attempted with glycemic control and tolerability taken into consideration. Patients undergoing diet and exercise therapy were treated with vildagliptin alone (addition group), and those undergoing treatment with hypoglycemic agents including insulin were switched to vildagliptin (switch group). After the start of vildagliptin therapy, the GA level and body weight were measured monthly, and clinical laboratory tests (mainly, blood and biochemical testes) were performed as needed.

### Statistical analysis

Long-term glycemic control by vildagliptin was evaluated based on the change in GA as the primary endpoint, and the GA level over time was compared between the 2 vildagliptin treatment groups. To detect potential modifiers of vildagliptin-induced changes in GA, single-regression and multiple-regression analyses were performed with the percentage change from baseline in GA at 2 years as the dependent variable; age, duration of diabetes, duration of dialysis, body mass index (BMI), and baseline GA were the independent variables.

The data referred showed that vildagliptin 50 mg qd reduced by 0.78 % in HbA1c from baseline Since the required number of patients was calculated as 21 to detect this difference with 1.2 in standard deviation under 5 % alpha levels and 80 % power. Thirty patients were considered to be required for 2-year evaluation.

Values were expressed as mean ± standard error, and changes in GA, dry weight, or BMI were subjected to analysis of variance (ANOVA) and multiple comparison versus baseline using Dunnett’s test. JMP 11 software (SAS Institute, Tokyo, Japan) was used for statistical analyses.

## Results

### Baseline characteristics and changes in antidiabetic therapy

The baseline characteristics of the 32 patients enrolled and followed up are shown in Table 1. They comprised 24 men and 8 women, aged 68.3 ± 1.9 years. Of the 32 patients, 9 received vildagliptin in addition to diet and exercise therapy alone, and 1 patient received vildagliptin in addition to voglibose (0.9 mg). The remaining 22 patients were switched from previous antidiabetic agents to vildagliptin, including 5 patients who were switched from insulin. The antidiabetic agents replaced with vildagliptin are listed in Table 2.

**Table 1:** Baseline patient characteristics

	*N* = 32
Male	24
Female	8
Age (years)	68.3 ± 1.9
BMI (kg/m^2^)	22.6 ± 0.6
Duration of diabetes (years)	20.0 ± 2.0
Duration of hemodialysis (months)	39.9 ± 6.9
GA level at baseline (%)	22.4 ± 0.6

BMI, body mass index; GA, glycated albumin. Values are mean ± standard error

**Table 2:** Medication before administration of vildagliptin

	*n*
Premedication	
No	9
Yes	23
Administration of vildagliptin	
Addition group	
Vildagliptin alone	9
Voglibose (0.9)	1
Switch group	
Insulin (4/8/16/18/25)	1/1/1/1/1
Glimepiride (0.5/2)	1/1
Voglibose (0.6/0.9)	1/3
Miglitol (50/150)	1/1
Mitiglinide (5/10/20/30)	1/2/1/1
Nateglinide (270)	1
Gliclazide (20) + voglibose (0.6)	1
Mitiglinide (30) + voglibose (0.9)	1
Nateglinide (60) + pioglitazone (30)	1

Numbers in parentheses are the dose of each agent (mg or IU)

During the 2-year follow-up, no patient was additionally treated with insulin, but the dose of vildagliptin was increased from 50 mg to 100 mg in 15 patients (5 in the addition group and 10 in the switch group). Of the 15 patients whose dose of vildagliptin was increased, 5 were additionally treated with mitiglinide (15 mg in 1 patient and 30 mg in 4 patients) and 1 was additionally treated with pioglitazone (30 mg). In 17 patients (5 in the addition group and 12 in the switch group), treatment with 50 mg of vildagliptin alone was maintained for 2 years.

### Glycemic control

**Fig. 1:**
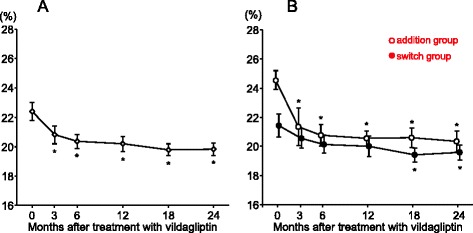
Changes in glycated albumin in all patients (**a**) and subgrouped patients (**b**). Open circles and closed circles in Fig. 1b indicate addition group and switch group, respectively. *p < 0.05 vs. baseline in Dunnett’s test. Values are mean ± standard error

GA over time in all patients is shown in Fig. 1a. GA was significantly reduced by 2.6 ± 0.6 %, from 22.4 ± 0.6 % at baseline (before vildagliptin therapy) to 19.8 ± 0.4 % at 2 years. In the addition group, GA was markedly reduced by 4.2 ± 0.8 %, from 24.5 ± 0.8 % at baseline to 20.3 ± 0.8 % at 2 years (Fig. 1b). GA was also significantly reduced from baseline in the switch group, but only by 1.9 ± 0.7 % at 2 years (Fig. 1b).

After 2 years of vildagliptin therapy, 15 (46.9 %) of 32 patients achieved a GA level of <20 %. GA was decreased in all 10 patients who had a GA of ≥24 % at baseline, and 8 of the 10 achieved a GA level of <24 % at 2 years.

In single-regression analysis of the percentage change in GA after 2 years of vildagliptin therapy in relation to age, duration of diabetes, duration of dialysis, baseline dry weight, and baseline GA, the change was positively correlated with the duration of dialysis (*r* = 0.46, *p* = 0.02) and negatively correlated with baseline GA (*r* = −076, *p* < 0.01, Table 3). In multiple-regression analysis of the percentage change in GA level at 2 years in relation to the duration of dialysis and baseline GA level, only baseline GA level was a significant factor (*p* < 0.01, Table 3).

**Table 3:** Single- or multiple-regression analysis

	Single analysis		Multiple analysis	
	*r*	*p* value	*β*	*p* value
Age (years)	−0.25	0.18		
BMI (kg/m^2^)	−0.25	0.16		
Duration of diabetes (years)	0.17	0.38		
Dry weight (kg)	−0.24	0.18		
Duration of hemodialysis (months)	0.46	0.02	0.15	0.28
GA level at baseline (%)	−0.76	<0.01	−0.73	<0.01

BMI, body mass index; GA, glycated albumin

### Change in dry weight and adverse reactions

**Fig. 2:**
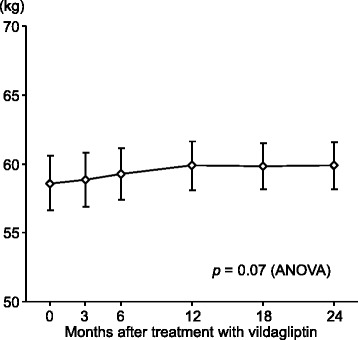
Changes in patient dry weight. Values are mean ± standard error

At the start of vildagliptin therapy, BMI in all patients was 22.6 ± 0.6 kg/m^2^, and dry weight was 58.8 ± 2.5 kg. Weight increased slightly over 2 years of vildagliptin therapy (Fig. 2), with an increase of 1.3 ± 0.8 kg at 2 years.

### Safety

No adverse drug reaction related to vildagliptin including abnormal changes on clinical examinations was noted during the 2-year treatment period.

## Discussion

In type 2 diabetic patients with ESRD, good glycemic control was maintained with little change in dry weight during 2-year vildagliptin therapy.

According to the Japan Diabetes Society, the target HbA1c level is <7.0 % for glycemic control to prevent complications of type 2 diabetes, and the goal should be glycemic normalization (HbA1c <6.0 %) if possible [12]. Many clinical studies showed that microangiopathy can be prevented with an HbA1c level of <7.0 %, and it is extremely challenging to achieve that target without weight gain or hypoglycemia. On the other hand, since few clinical studies have been conducted in type 2 diabetic patients with ESRD, no target glycemic control has been defined for them, although strict glycemic control may be required given complications such as retinopathy or neuropathy.

Vildagliptin was chosen as the current study drug because only two DPP-4 inhibitors, sitagliptin and vildagliptin, were available in Japan when this study was planned. It was believed that vildagliptin was more suitable for type 2 diabetic patients with ESRD than sitagliptin by comparing their pharmacokinetics in patients with renal failure [8, 13], and two vildagliptin doses were available, indicating that dose adjustment would be possible.

DPP-4 inhibitors, which have been shown in meta-analysis to be associated with a low incidence of hypoglycemia and little risk of weight gain [14, 15], are useful to achieve therapeutic targets. In addition, while antidiabetic agents available for patients with ESRD are limited, some DPP-4 inhibitors can be administered if the dose is adjusted. Vildagliptin, alogliptin, and sitagliptin have been studied in type 2 diabetic patients with ESRD.

In a study of alogliptin, insulin-free patients receiving or not receiving treatment with mitiglinide or voglibose were additionally treated with alogliptin (*n* = 30) to evaluate efficacy and safety over 48 weeks, and it was found that HbA1c and GA levels were significantly reduced [16]. In another study, patients received alogliptin for 2 years after withdrawal from previous antidiabetic agents (*n* = 13), and both HbA1c and GA levels were reduced [17]. In a double-blind study of sitagliptin versus sulfonylurea (SU), patients received the study drug for 54 weeks after withdrawal from previous antidiabetic agents (*n* = 64), and both drugs significantly reduced HbA1c levels [18]. However, the incidence of adverse hypoglycemic events tended to be higher with SU, and serious hypoglycemia was reported in 7.7 % of patients treated with SU but not in those treated with sitagliptin. Two studies of vildagliptin administered for 24 weeks (*n* = 30) [19] and 6 months (*n* = 15) [20] were reported. Both were open-label studies of additional treatment with vildagliptin versus continued existing drugs and confirmed the usefulness of vildagliptin.

Since the treatment of type 2 diabetes continues over time, the long-term safety and efficacy should be evaluated. For the present study, therefore, 2-year follow-up was selected. Moreover, previous antidiabetic agents, including insulin, were replaced with vildagliptin to avoid the risk of hypoglycemia, although vildagliptin in addition to previous drugs may be more effective. These conditions were different from those in previous reports, but the dose of vildagliptin was increased to 50 mg twice daily in 15 (46.9 %) of 32 patients during the 2-year follow-up. While the dose increase resulted in a 0.8 % reduction in GA, 5 patients were additionally treated with mitiglinide or pioglitazone, suggesting that glycemic control cannot be achieved by vildagliptin alone at a dose of 50 mg once daily in many patients. However, since hypoglycemia did not occur after the dose increase or addition of other oral antidiabetic agents, more aggressive treatment may be possible.

Since GA is highly correlated with the HbA1c level and is approximately 3-fold the HbA1c level [21], the Japanese Society for Dialysis Therapy recommends a target GA level of <20 % for glycemic control in type 2 diabetes with ESRD as a temporary goal and a target GA level of <24 % in patients with a history of cardiovascular disease or a risk of hypoglycemia [22]. In the present study, a GA level of <20 % was maintained in 46.9 % of patients after 2 years of vildagliptin therapy, and a reduction of GA at 2 years was found in all patients with GA ≥24 % at baseline, showing the efficacy of vildagliptin even though it replaced previous antidiabetic agents.

## Conclusions

In type 2 diabetic patients undergoing hemodialysis, vildagliptin-based therapy for 2 years maintained lower blood glucose level measured by GA and showed no weight gain and hypoglycemia. Vildagliptin is considered a promising therapeutic option for type 2 diabetic patients with ESRD.
